# LEVELING THE PLAYING FIELD: ONBOARDING AGING FELLOWS REPRESENTING DIVERSE DISCIPLINES

**DOI:** 10.1093/geroni/igac059.728

**Published:** 2022-12-20

**Authors:** Catherine Kim, Nia Latimer, Katherine Bowers, Loretta Anderson, Diane Martin

**Affiliations:** Veterans Affairs Maryland Health Care System, Baltimore, Maryland, United States; University of Maryland, Baltimore, Baltimore, Maryland, United States; University of Maryland, Baltimore, Maryland, United States; University of Maryland, Baltimore, Reisterstown, Maryland, United States; University of Maryland, Baltimore Graduate School, Baltimore, Maryland, United States

## Abstract

The transdisciplinary Innovations in Aging Graduate Fellowship program brought together graduate students from medicine, nursing, pharmacy, and social work. As a result, fellows entered the program with a range of exposure as to how aging impacts the body, social interactions, health needs, and society. Therefore, onboarding modules were utilized to provide an integrated perspective on aging. Topics for these onboarding modules included ageism, dimensions of wellness, and health and well-being in later life. Through these modules, fellows were able to customize their learning to meet their unique educational needs and goals related to their involvement in the fellowship program. In this session, we will share development of the fellowship program and highlight factors, such as the onboarding modules, that led to the program’s success.

